# The Role of Non-Curative Surgery in Incurable, Asymptomatic Advanced Gastric Cancer

**DOI:** 10.1371/journal.pone.0083921

**Published:** 2013-12-16

**Authors:** Ming-ming He, Dong-sheng Zhang, Feng Wang, Zhi-qiang Wang, Hui-yan Luo, Ying Jin, Xiao-li Wei, Rui-hua Xu

**Affiliations:** 1 State Key Laboratory of Oncology in South China, Guangzhou, China; 2 Department of Medical Oncology, Sun Yat-sen University Cancer Center, Guangzhou, China; Sun Yat-Sen University Cancer Center, China

## Abstract

**Background:**

Although general agreement exists on palliative surgery with intent of symptom palliation in advanced gastric cancer (AGC), the role of non-curative surgery for incurable, asymptomatic AGC is hotly debated. We aim to clarify the role of non-curative surgery in patients with incurable, asymptomatic AGC under the first-line chemotherapy.

**Methods:**

A total of 737 patients with incurable, asymptomatic advanced gastric adenocarcinoma between January 2008 and May 2012 at the Sun Yat-sen University Cancer Center were retrospectively analyzed, comprising 414 patients with non-curative surgery plus first-line chemotherapy, and 323 patients with first-line chemotherapy only. The clinicopathologic data, survival, and prognosis were evaluated, with propensity score adjustment for selection bias.

**Results:**

The median overall survival (OS) outcomes significantly favored non-curative surgery group over first-line chemotherapy only group in entire population (28.00 versus 10.37 months, P = 0.000), stage 4 patients (23.87 versus 10.37 months, P = 0.000), young patients (28.70 versus 10.37 months, P = 0.000) and elderly patients (23.07 versus 10.27 months, P = 0.031). The median OS advantages of non-curative surgery over first-line chemotherapy only were also maintained when the analyses were restricted to single organ metastasis (P = 0.001), distant lymph node metastasis (P = 0.002), peritoneal metastasis (P = 0.000), and multi-organ metastasis (P = 0.010). Significant OS advantages of non-curative surgery over chemotherapy only were confirmed solid by multivariate analyses before and after adjustment on propensity score (P = 0.000). Small subsets of patients with surgery of single metastatic lesion after previous curative gastrectomy, and with surgery of both primary and single metastatic sites showed sound median OS.

**Conclusions:**

There is a role for non-curative surgery plus first-line chemotherapy for incurable, asymptomatic AGC, in terms of survival. Randomized controlled trials are warranted to fill a gap in knowledge about the value of metastectomy and patient selection strategies.

## Introduction

Gastric cancer ranks second among the most common causes of cancer deaths worldwide, with especial high prevalence in countries of northeast Asia [1]. The majority of gastric cancer patients present with locally advanced, recurrent or metastatic disease precluding curative surgery and usually receives non-curative therapy [2], [3]. Evidently, palliative chemotherapy evolves as the primary management strategy for advanced gastric cancer (AGC) patients [4].

While general agreement exists that surgery is indicated to palliate the major symptoms such as bleeding or obstruction in AGC [5], the clinical value in non-curative surgical management of patients with minimal symptoms and incurable disease is debated [6]. The Japanese Gastric Cancer Association (JGCA) guidelines indicate that patients with metastases may be candidates for gastrectomy without major symptoms [7], however, the National Comprehensive Cancer Network (NCCN) only recommends patients with symptoms as candidates for surgery. There is still insufficient evidence to recommend non-curative resection in terms of survival benefit achieved in retrospective studies which demonstrated controversial results [8]–[11], had variable understanding of the indications and intents of non-curative surgery [5], [12]–[16], failed to balance the palliative chemotherapy during comparison [9], [17], [18] and confused the influence of disease stage, tumor load and other baseline clinical factors [19], [20].

Therefore, this retrospective study was designed to clarify the role of non-curative surgery in patients with incurable, asymptomatic AGC under the first-line chemotherapy and provide information for clinicians weighing multiple factors before decision-making.

## Patients and Methods

### Ethics Statement

This study was approved by the Institution Review Board of Sun Yat-sen University Cancer Center. First-line treatment and retrospective analysis of medical records were performed after obtaining written informed consent from all patients and approval from the independent Institute Research Ethics Committee at the Cancer Center of Sun Yat-sen University. We conducted this retrospective research according to the principles expressed in the Declaration of Helsinki.

### Patients

Between January 2008 and May 2012, a total of 737 patients were histologically proven and diagnosed as incurable, asymptomatic advanced gastric adenocarcinoma in Sun Yat-sen University Cancer Center and received first-line chemotherapy. Among them, 414 patients also underwent non-curative surgery which comprised 395 patients with non-curative gastrectomy, 14 patients with palliation of metastatic lesion and 5 patients with both before, during or after first-line chemotherapy, while 323 patients had first-line chemotherapy only. We reviewed the medical records of all 737 patients and unified the staging according to American Joint Committee on Cancer (AJCC, seventh edition).

The inclusion criteria for non-curative surgery group were: (1) patients with metastatic gastric cancer who underwent non-curative surgery and then received first-line chemotherapy; (2) patients with metastatic gastric cancer who achieved partial-response or stable disease after several cycles of first-line chemotherapy and then had non-curative surgery whether they continued first-line chemotherapy or not after the surgery; (3) patients who presented with recurrence (broadly judged as stage 4) or metastasis after previously curative gastrectomy and then had non-curative surgery, first-line chemotherapy; (4) stage 3 patients with locally advanced gastric cancer who had non-curative gastrectomy or R2 gastrectomy and then had first-line chemotherapy.

The inclusion criteria for first-line chemotherapy only group: (5) patients who presented with recurrence or metastasis after previously curative gastrectomy and had first-line chemotherapy; (6) patients with metastatic gastric cancer who had first-line chemotherapy.

Baseline evaluation included medical history, physical examination, Charlson score, complete blood count, serum chemistry, serum tumor markers, electrocardiography, imaging and pathological examination. All regular follow-up assessments were completed by July 20^th^, 2013. The median follow-up was 35.0 months (range 0.1 to 66.5).

### Statistical Analysis

The chi-square test was used to compare categorical variables between the non-curative surgery group and the first-line chemotherapy only group. Nonparametric tests were used to compare continuous variables. Overall survival (OS) was calculated from the initiation of first-line treatment (either non-curative surgery or first-line chemotherapy) to death from any cause. Unadjusted Kaplan-Meier survival curves with log rank testing were generated to compare the survival benefits between treatment groups. Prognostic factors were analyzed by searching clinicopathological factors in univariate analysis, with all variables with a P value < 0.05 in the univariate analysis entered into multivariate analysis using Cox proportional hazard regression models. The hazard ratio (HR) and 95% confidence interval (CI) were used to estimate the role of each predictor of survival. A two-sided P value < 0.05 was considered significant. All statistical analyses were performed using the SPSS software (version 19.0, SPSS, Chicago, IL, USA).

### Propensity Score Analysis

A propensity score was built and used as adjustment variable to control for selection bias in this no randomized study. The propensity score, which represents the conditional probability of receiving a therapy given a vector of covariates, is commonly used in observational studies to adjust for selection bias [21].

A logistic regression model was used to estimate the propensity score (probability of receiving first-line chemotherapy only) for each of the 737 patients. Covariates that may influence both the treatment selection and the survival were included in the model, which were age, sex, Charlson score, AJCC stage, tumor location, histological differentiation, tumor size, baseline serum tumor markers, previous radical gastrectomy, and second-line chemotherapy. The model showed 73% of correctly classified patients. A propensity score for stage 4 subpopulation was also performed with the same included covariates except AJCC stage, with 61% of correctly classified patients. Patients were then assigned to 4 strata based on the estimated propensity score; each stratum contained 25% of the patients. Multivariate analyses of the entire population and the stage 4 subpopulation were adjusted for the propensity score in 4 strata [22].

## Results

### Patient Characteristics

The non-curative surgery group comprised 190 stage 3 patients and 224 stage 4 patients, while the first-line chemotherapy only group comprised 323 stage 4 patients. The clinicopathological characteristics for both groups in entire population and stage 4 subgroup were summarized in Table 1 and Table 2, respectively.

**Table 1:** Clinicopathological characteristics of patients with advanced gastric cancer.

Characteristics	Non-curative surgery + first-line chemotherapy	First-line chemotherapy only	P
No. of patients	414	323	
Age(years, median, range)	55(23–84)	55(19–84)	0.988
< 70 year	378(91.3)	298(92.3)	0.640
≥ 70 year	36(8.7)	25(7.7)	
Sex, n			0.175
Men	280 (67.6)	203 (62.8)	
Women	134 (32.4)	120 (37.2)	
Charlson score			0.354
0	5(1.2)	4( 1.1)	
1	404(97.6)	300 (93)	
2	5(1.2)	19 (5.9)	
Tumor location			0.265
Proximal	275 (66.4)	227 (70.3)	
Distal	139 (33.6)	96 (29.7)	
Histological grade			0.252
High-differentiation	5(1.2)	7(2.2)	
Moderate-differentiation	51(12.3)	49(15.2)	
Low-differentiation	267(64.5)	211(65.3)	
Poor-differentiation	91(22.0)	56(17.3)	
Gloss type			0.118
Protrusion	87 (21.0)	89 (27.6)	
Ulcer	265(64.0)	190 (58.8)	
Infiltration	62 (15.0)	44 (13.6)	
Size			0.625
<5 cm	195(47.1)	158(48.9)	
≥5 cm	219(52.9)	165(51.1)	
Serum CEA(ng/ml, median, range)	2.4(0.2–6576.0)	3.6(0.3–7617.0)	0.008
Serum CA19-9(U/ml, median, range)	12.6(0.6–12210.0)	28.1(0.6–20000.0)	0.000
Serum CA72-4(U/ml, median, range)	2.8(0.6–1298.0)	7.9(0.7–1425.0)	0.000
Hemoglobin(g/l, median, range)	120.2(78–168)	122.6(83–162)	0.522
Albumin(g/l, median, range)	38.8(24.9–49.0)	38.3(17.3–54.0)	0.306
ALT(U/L, median, range)	16.8(3.4–87.0)	15.9(0.9–88.3)	0.270
AST(U/L, median, range)	19.5(10.3–90.9)	20.2(6.1–93.3)	0.235
Tbil(mmol/l, median, range)	9.0(2.8–28.0)	9.5(2.6–29)	0.520
Ascites			0.000
Yes	17(4.1)	53(17.0)	
No	396(95.9)	270(83.0)	
Second-line chemotherapy			0.000
Yes	121(29.2)	144(44.6)	
No	293(70.8)	179(55.4)	

**Abbreviations:** CEA, baseline carcinoembryonic antigen; CA19-9, baseline carbohydrate antigen 19-9; CA72-4, baseline carbohydrate antigen 72-4; ALT, baseline alanine aminotransferase; AST, baseline aspartate aminotransferase; Tbil, baseline total bilirubin.

**Table 2:** Clinicopathological characteristics of patients with stage 4 gastric cancer.

Characteristics	Non-curative surgery + first-line chemotherapy	First-line chemotherapy only	P
No. of patients	223	323	
Age (years, median, range)	55(23–84)	55(19–84)	0.928
<70 year	205(91.5)	298(92.3)	0.856
≥70 year	19(8.5)	25(7.7)	
Sex, n			0.378
Men	149(66.5)	203 (62.8)	
Women	75 (33.5)	120 (37.2)	
Charlson score			0.354
0	4(1.8)	4( 1.1)	
1	215(96.4)	300 (93)	
2	4(1.8)	19 (5.9)	
Tumor location			0.208
Proximal	146 (65.2)	227 (70.3)	
Distal	78 (34.8)	96 (29.7)	
Histological grade			0.273
High-differentiation	5(2.2)	7(2.2)	
Median-differentiation	33(14.7)	49(15.2)	
Low-differentiation	132(58.9)	211(65.3)	
Poor-differentiation	54(24.1)	56(17.3)	
Gloss type			0.130
Protrusion	56 (25.0)	89 (27.6)	
Ulcer	123(54.9)	190 (58.8)	
Infiltration	45 (20.1)	44 (13.6)	
Size			0.284
<5 cm	120(53.6)	158(48.9)	
≥5 cm	104(46.4)	165(51.1)	
Serum CEA(ng/ml, median, range)	2.6(0.2–566.5)	3.6(0.3–7617.0)	0.021
Serum CA19-9(U/ml, median, range)	14.3(0.6–12210.0)	28.1(0.6–20000.0)	0.000
Serum CA72-4(U/ml, median, range)	3.5(0.6–1298.0)	7.8(0.7–1425.0)	0.001
Hemoglobin(g/l, median, range)	118.0(78–164)	122.6(83–162)	0.166
Albumin(g/l, median, range)	38.3(24.9–49.0)	38.3(17.3–54.0)	0.879
ALT(U/L, median, range)	16.7(3.4–87.0)	15.9(0.9–88.3)	0.724
AST(U/L, median, range)	19.4(10.5–90.9)	20.2(6.1–93.3)	0.158
Tbil(mmol/l, median, range)	8.8(2.8–27.2)	9.5(2.6–29)	0.377
Ascites			0.001
Yes	17(7.6)	55(17.0)	
No	206(92.4)	268(83.0)	
Second-line chemotherapy			0.079
Yes	83(37.1)	144(44.6)	
No	141(62.9)	179(55.4)	

**Abbreviations:** Stage 4, including metastatic and recurrent gastric cancer; CEA, baseline carcinoembryonic antigen; CA19-9, baseline carbohydrate antigen 19-9; CA72-4, baseline carbohydrate antigen 72-4; ALT, baseline alanine aminotransferase; AST, baseline aspartate aminotransferase; Tbil, baseline total bilirubin.

### Survival

**Figure 1:**
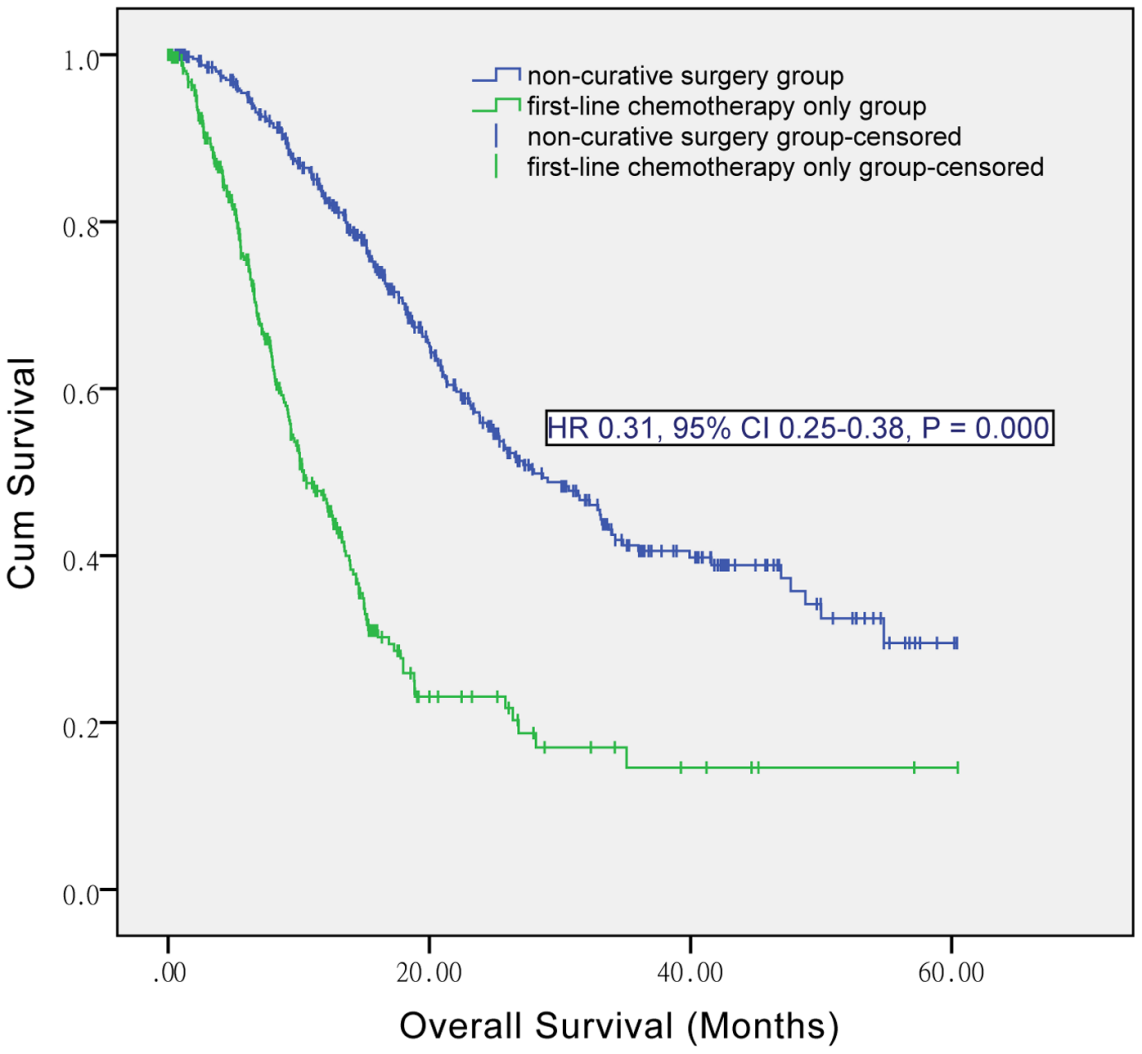
Kaplan-Meier curves of non-curative surgery group and first-line chemotherapy only group, in entire population. HR, hazard ratio; CI, confidence interval.

The median OS was significantly higher in non-curative surgery group than in first-line chemotherapy only group (28.00 [95% CI 23.22–32.78] versus 10.37 [8.57–12.18] months; P = 0.000) (Figure 1).

**Figure 2:**
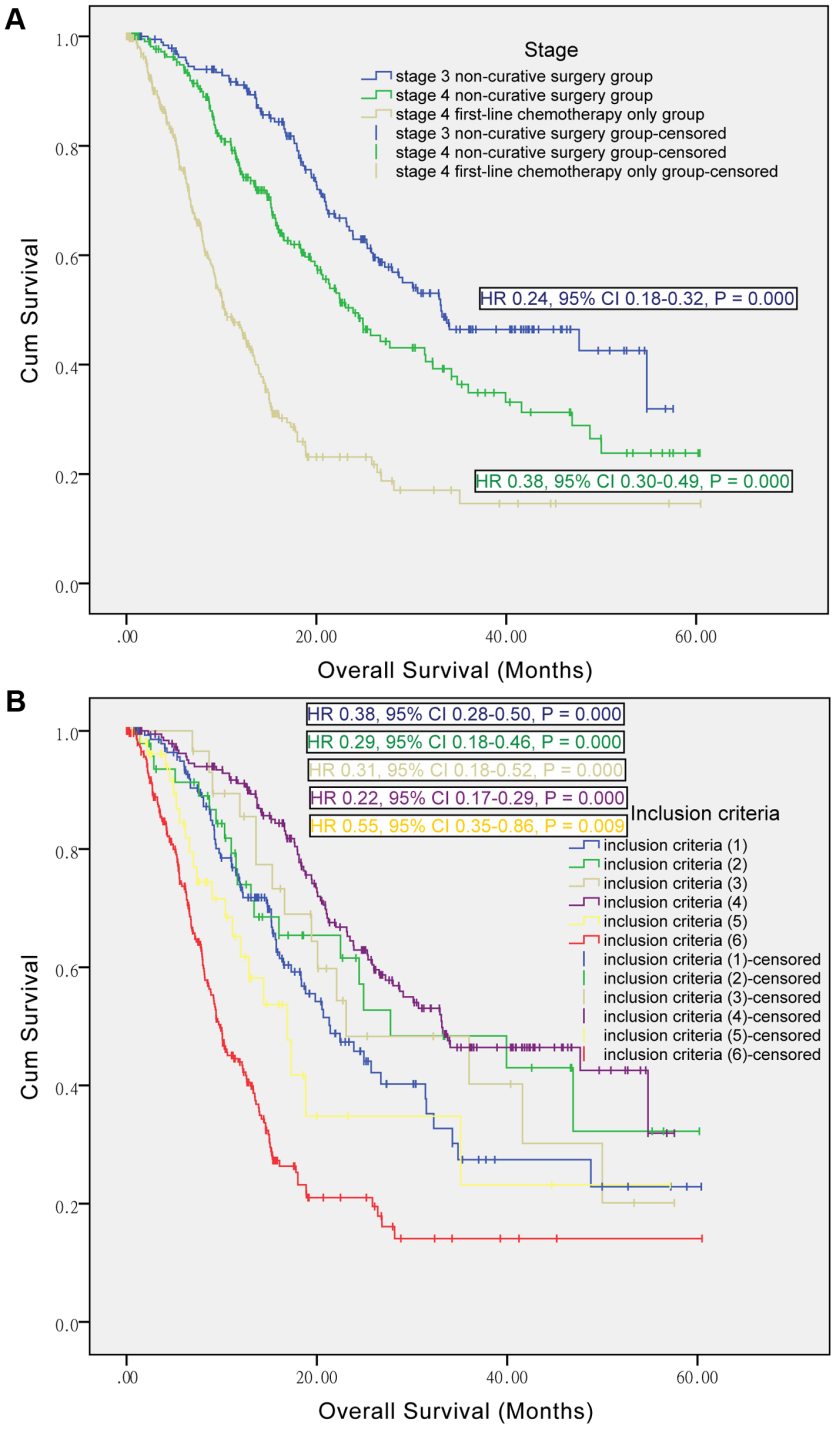
Kaplan-Meier curves of non-curative surgery and first-line chemotherapy only groups, by stages and inclusion criteria. Figure 1A shows subgroup analysis according to stage, with stage 4 first-line chemotherapy only group as reference. Stage 4 here included metastatic and recurrent gastric cancer. Figure 1B shows subgroup analysis according to inclusion criteria, with the inclusion criteria (6) as reference. HR, hazard ratio; CI, confidence interval.

**The role of non-curative surgery according to AJCC stage.** In the subgroup of stage 4 patients, the median OS outcome still significantly favored non-curative surgery group over first-line chemotherapy only group (23.87 [19.56–28.18] versus 10.37 [8.57–12.18] months; P = 0.000), as shown in Figure 2A. This figure also demonstrated significant longer median OS for stage 3 patients of non-curative surgery group over first-line chemotherapy only group (33.13 [20.73–45.53] versus 10.37 [8.57–12.18] months; P = 0.000).

**The role of non-curative surgery according to inclusion criteria.** Both groups included several subpopulations as shown in the inclusion criteria. Figure 2B presented the survival curves of these subpopulations. The median OS for inclusion criteria (1) (2) (3) (4) (5) (6) were 21.40 [16.04–26.76], 27.73 [7.85–47.61], 23.07 [5.62–40.52], 33.13 [20.73–45.53], 16.90 [11.79–22.01] and 9.80 [8.29–11.31] months, respectively. We set the patients with metastatic gastric cancer who received first-line chemotherapy only (namely the inclusion criteria (6)) as reference, and then found the median OS for the reference was significantly lower than other inclusion criteria ((1) P = 0.000, (2) P = 0.000, (3) P = 0.000, (4) P = 0.000 and (5) P = 0.009).

**Figure 3:**
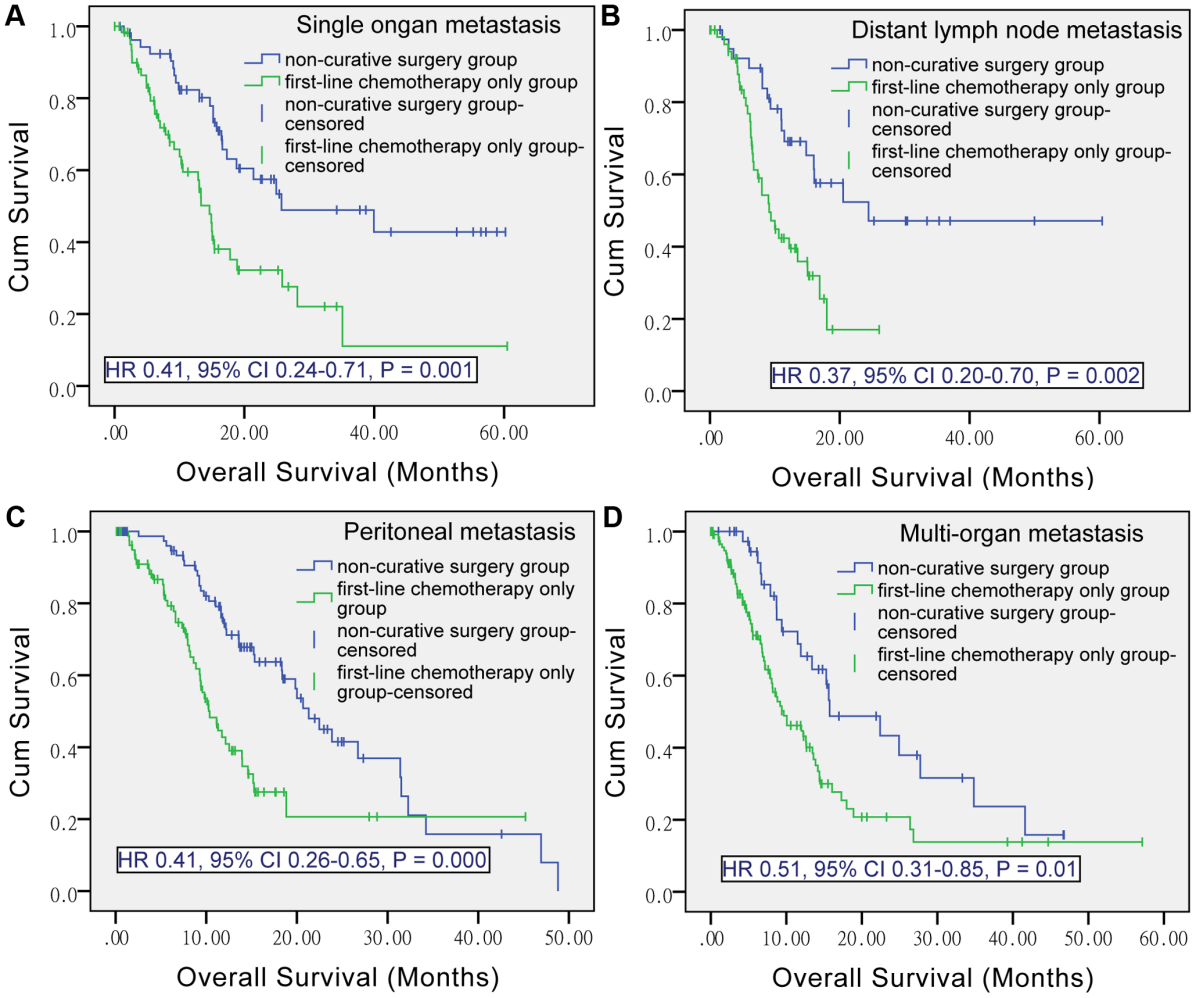
Kaplan-Meier curves of non-curative surgery group and first-line chemotherapy only group, by metastasis types. HR, hazard ratio; CI, confidence interval.

**The role of non-curative surgery according to metastasis types.** These median OS advantages of the non-curative surgery over the first-line chemotherapy only were maintained when the analyses were restricted to single organ metastasis (N, 54 versus 63; 25.70 versus 14.63 months; P = 0.001), distant lymph node metastasis (N, 39 versus 54; 24.43 versus 9.13 months; P = 0.002), peritoneal metastasis (N, 82 versus 81; 21.30 versus 10.37 months; P = 0.000), and multi-organ metastasis (N, 40 versus 121; 15.73 versus 9.67 months, P = 0.010) (Figure 3).

**The role of non-curative surgery according to surgery types.** Different surgery types included non-curative gastrectomy, palliation of metastatic lesion and both of them. Minimizing the effect of disease stage, we restricted to stage 4 patients. The median OS for non-curative gastrectomy, palliation of metastatic lesion and both of them were 22.47 [18.71–26.24], 50.00 [16.31–83.69] and 46.93 [0.00–107.73] months, with significant difference (P = 0.000, P = 0.010, P = 0.047) compared to that for the first-line chemotherapy only (10.37 [8.57–12.18] months). However, there were no significant differences between any two of these surgery types (P = 0.454, P = 0.674, P = 0.647) (See Figure S1).

**The role of non-curative surgery according to patient age.** These median OS advantage of the non-curative surgery over the first-line chemotherapy only were maintained in young patients with age < 70y (28.70 [23.97–33.43] versus 10.37 [8.55–12.19] months; P = 0.000) and elderly patients with age ≥ 70y (23.07 [12.85–33.29] versus 10.27 [2.58–17.96] months; P = 0.031) (See Figure S2).

### Prognostic Factors

Univariate analysis (See Table S1) and multivariate analysis (See Table S2) in the entire population showed the non-curative surgery plus first-line chemotherapy, AJCC stage 3, distal tumor location, no baseline ascites, and baseline serum carcinoembryonic antigen (CEA) < median were independent prognostic factors for prolonged OS. After adjusting for the 4 propensity score strata, these 5 factors were still independently associated with OS (Table 3). In stage 4 subgroup, the non-curative surgery plus first-line chemotherapy, no baseline ascites, baseline serum CEA < median, baseline serum carbohydrate antigen 19-9 (CA19-9) < median were independent prognostic factors for prolonged OS (See Table S3). After adjusting for the 4 propensity score strata, these 4 factors were still independently associated with OS, while the second-line chemotherapy was also independently associated with prolonged OS (Table 4). Meanwhile, distributions of the propensity score according to treatment group in entire population and stage 4 subpopulation were given (See Figure S3 and Figure S4). Figure S3 showed similar distributions between the two treatment groups, except that the stage 3 patients would only be classified to non-curative group (propensity score was zero). Figure S4 showed similar distributions between the two treatment groups in stage 4 subpopulation, after excluding stage 3 patients.

**Table 3:** Multivariate analysis of overall survival in patients with advanced gastric cancer after adjustment on the propensity score.

Variate	HRa	95% Cl	P
Treatment			0.000
Non-curative surgery+chemotherapy	0.36	0.26–0.51	
Chemotherapy only	1	reference	
AJCC stage			0.040
Stage 3	0.46	0.22–0.96	
Stage 4	1	reference	
Tumor location			0.026
Proximal	1.44	1.05–1.98	
Distal	1	reference	
Ascites			0.023
No	0.50	0.28–0.91	
Yes	1	reference	
Serum CEA			0.044
< the median	0.74	0.56–0.99	
≥ the median	1	reference	
Propensity score	0.87	0.59–1.26	0.453

**Abbreviations:** HRa, hazard ratio adjusted on the propensity score in 4 strata; CI, confidence interval; AJCC, American Joint Committee on Cancer; Stage 4, including metastatic and recurrent gastric cancer; CEA, baseline carcinoembryonic antigen.

**Table 4:** Multivariate analysis of overall survival in patients with stage 4 gastric cancer after adjustment on the propensity score.

Variate	HRa	95% Cl	P
Treatment			0.000
Non-curative surgery+chemotherapy	0.36	0.25–0.51	
Chemotherapy only	1	reference	
Ascites			0.022
No	0.57	0.35–0.92	
Yes	1	reference	
Serum CEA			0.033
< the median	0.68	0.48–0.97	
≥ the median	1	reference	
Serum CA19-9			0.032
< the median	0.68	0.48–0.97	
≥ the median	1	reference	
Second-line chemotherapy			
No	1.46	1.02–2.10	0.037
Yes	1	reference	
Propensity score	1.21	0.81–1.80	0.349

**Abbreviations:** Stage 4, including metastatic and recurrent gastric cancer; HRa, hazard ratio adjusted on the propensity score in 4 strata; CI, confidence interval; CEA, baseline carcinoembryonic antigen; CA19-9, baseline carbohydrate antigen 19-9.

## Discussion

Non-curative surgery in AGC mainly included two categories according to intents; the palliative surgery with intent of symptom palliation had been generally accepted, however, whether non-curative surgery is worthwhile for patients with incurable, asymptomatic disease with intent of prolonging overall survival is hotly debated. Thus, the most suitable index for our study is OS and significant OS advantages of non-curative surgery over first-line chemotherapy only was found, which were confirmed solid by multivariate analyses before and after adjustment on the propensity score.

Actually, the median survival of non-curative surgery varied by treatment intents [9]. For asymptomatic patients undergoing non-curative surgery with no intent of symptom palliation, the median OS ranged from 5 to 24 months [17], [20], [23]–[28]. The non-curative surgery with the intent of symptom palliation, which usually included resectional surgery and non-resectional surgery such as surgical bypass, achieved a narrow range of median OS from 3 to 13 months [18], [29]–[32]. Evidence showed the significantly superior survival prognosis of the non-palliative, non-curative resection than palliative, non-curative resection [12] and palliative bypass [33], [34]. In comparison to many studies with mixed intents, one important reason for this long median OS (28 months) of non-curative surgery group is that we centered on non-palliative, non-curative resections for asymptomatic patients in most recent years.

Most previous studies have mixed different disease stages when analyzing non-curative procedures, which caused hot debates. The prognosis after resection depends on the pathologic stage with evidence from previous literature and also the multivariate analysis in our results [24]. The proportion of stage 4 disease in the previous study populations ranged from 12 to 100%. The Samarasam et al published long median OS as 24 months, included 77.4% stage 4 disease, 14.6% stage 3 for entire sample and the stage distribution for the surgery group was unknown [20]. In our current series, we included 190 (45.9%) stage 3 patients, 224 (54.1%) stage 4 patients in the non-curative surgery group and 323 stage 4 patients in chemotherapy only group. This subset of stage 3 patients initially were to have curative-intent surgery, however had non-curative resection with macroscopically positive margin determined intra-operatively, which got a median OS of 33.13 months. Previous literature showed most favorable OS for these initially curative intent patients (median OS 7 to 33.9 months) and supported gastrectomy for local advanced gastric cancer [10], [12], [34]–[37], in accord to our finding.

Controversy with conflicting conclusions on the benefit of surgery for stage 4 disease existed across previous reports. Firstly, the attitude about the gastrectomy for stage 4 disease is controversial. Our results showed non-curative surgery achieved significant survival benefit over chemotherapy only in stage 4 patients (median OS, 23.87 versus 10.37 months), in general accord with most published series of non-curative gastrectomy showing significant improvement in survival and quality of life [2], [9], [18], [24], [38], and in contrast with some studies showing no survival benefit, or worse quality of life [8], [39]. Based on this situation many but not all authors proposed that primary resections should be performed whenever technically possible and patients with metastasis may be candidates for gastrectomy was recommended by JGCA guidelines [7], [39], however, not by NCCN. Secondly, feasibility for different metastasis is discussed. We found consistent significant OS advantage of non-curative surgery over first-line chemotherapy only in single organ metastasis, distant lymph node metastasis, peritoneal implantation, and multi-organ metastasis (in descending sort of median OS). Based on the survival benefits, feasibility of non-curative gastrectomy for single organ metastasis, distant lymph node metastasis was generally accepted by many authors [10], [38]. Evidence existed of improved survival by non-curative gastrectomy in peritoneal metastatic disease, with the median OS ranging from 5 to 21.7 months [9], [38], [40]–[43]. However, the opposite evidence of gastrectomy or bypass in peritoneal metastasis also existed [10], [44] and authors failed to build consensus on the feasibility of surgery. One reason is a wide range with respect to involved area, number and size of peritoneal tumors [45]. The peritoneal carcinosis score published by Jacquet P and Sugarbaker PH helps to classify peritoneal metastasis into P1, P2 and P3 [46]. Literature showed P1 was indicated for non-curative gastrectomy, while P2, P3 were not [24]. In our study, most of the included peritoneal implantation cases were judged as P1, which explain the relative better median OS than previous reported. Much work should be done to select patients with peritoneal metastasis for gastrectomy, especially with respect to the impact of novel perioperative chemotherapy [9], [47]. The value of gastrectomy in multi-organ metastasis is uncertain. The Dutch Gastric Cancer Group suggested that differences in overall survival after non-curative gastric resections may be beneficial in patients with tumor load restricted to one metastatic site [48]. There’re evidence that the survival difference and the resectability decreased with the increasing metastatic sites [20]. The value of gastrectomy in multi-organ metastasis awaits more investigation. Thirdly, whether the metastatic lesion should be reduced and the range of surgery remained unknown. In our study, 414 patients underwent non-curative surgery comprised 395 patients with non-curative gastrectomy, 14 patients with only palliation of metastatic lesion and 5 patients with both. All the 14 patients had one metastatic site after previously curative gastrectomy, which was then resected. This strategy is reasonable and achieved the sound median OS. The 5 patients with resection of both primary and the only one metastatic sites achieved sound median OS, too. Although no vital complications were observed in these 5 patients, some literature showed combined resection were closely related to postoperative complications in patients with non-curative gastrectomy [49]. This strategy is not so commonly applied and the value and safety awaits investigation. The ongoing GYMSSA and REGATTA trials which evaluate the survival benefit and adverse events associated with gastrectomy with metastectomy and systemic therapy versus systemic therapy alone in metastatic gastric cancer patients [50], [51], are expected to highlight this question.

Chemotherapy is an independent factor for prolonged survival in AGC patients with or without non-curative surgery [9], [17]. The OS is disappointing for non-curative gastric resection without chemotherapy. With administration of chemotherapy pre- and postoperatively, the survival time increased obviously [9], [52]. The above reasons help to explain the relative longer median OS in our study than previous literatures which neither illustrated the chemotherapy status of the patients nor gave chemotherapy to all patients [9], [10], [18]. The median OS of the chemotherapy only group (10.37 months) here is the average level treated with palliative chemotherapy for AGC. The synergetic effects of non-curative resection and chemotherapy included improved chemotherapy sensitivity of residual tumor after resection, less immunosuppressive factor release, and reduction of tumor stem cells with the tumor resection [9], [53]. That helped explain why the non-curative surgery group achieved long median OS on the ground of chemotherapy only. In subgroup analysis according to inclusion criteria, we found sound survival for preoperative chemotherapy (inclusion criteria (2)), and especially surgery plus both pre- and postoperative chemotherapy yield the best prognosis, although the difference wasn’t significant. We expect randomized controlled trial comparing different timing of non-curative surgery with chemotherapy based on the promising finding of this small subset as inclusion criteria (2), with more patients and enough statistic power. To explore the best drug partner of non-curative surgery, we further accessed the regimen and found platinum-containing chemotherapy or not, single drug or combination chemotherapy was not prognostic for OS. What’s more, the best evaluation of chemotherapy efficacy was not prognostic of OS. The choice of ideal chemotherapy before and after non-curative surgery remained to be investigated.

Another important issue is patient selection for first-line chemotherapy only or plus non-curative surgery and the prognosis of the factors. Clinicopathological characteristics were balanced except the baseline serum CEA, CA19-9, carbohydrate antigen (CA 72-4), ascites, and second-line chemotherapy. In stage 4 subgroup, second-line chemotherapy was again balanced. Although the primary tumor size was comparable, the differences of tumor markers and ascites mean the patients who receive non-curative surgery, compared with those who receive first-line chemotherapy only, likely have a lower burden of disease, in accord with previous literature [6]. The serum CEA, serum CA19-9, and ascites were borderline independent prognostic factors revealed by the multivariate analysis here. This difference reflected the clinical thinking, stratification and selection by surgeons, as well as confused the evaluation of treatment. Thus we did subgroup analyses according to baseline tumor marker level (< median, ≥ median) and ascites (yes, no), and still found significant difference of OS between the two treatment groups (See Figure S5 and Figure S6). To better overcome the imbalance of patients’ characteristics, some compounding factors and their poteintal confusion of survival difference, propensity score analyses were performed. Multivariate analyses of the entire population and the stage 4 subpopulation before and after adjustment on propensity score showed consistent results of independent prognostic factors, in which the advantages of non-curative surgery over first-line chemotherapy only were always confirmed robust. Of note, second-line chemotherapy emerged independently prognostic of prolonged survival in the stage 4 subpopulation after adjustment on the propensity score. The role of second-line chemotherapy has been suggested previously. In our center, the second-line chemotherapy assignment to non-curative surgery was significantly less than to first-line chemotherapy only group in entire population and relative less in stage 4 patients. This fact strengthened the survival benefit of non-curative surgery group. Yet, whether it is for patients with non-curative surgery may have progression disease later than chemotherapy only, or for patients’ own choice remains to be investigated. In contrast with many studies selecting young patients for non-curative surgery, the age is well balanced in our study. Non-curative surgery yielded survival benefit in both young and elderly patients, comparable to previous reports and thus many authors held age was not a limiting factor [49], [54]. Some authors worried about the higher surgical morbidity and mortality for old patients and suggested more attention be paid to the perioperative care [55]. Randomized controlled trials are warranted to fill a gap in knowledge about patient selection strategies.

The limitations of this study are the retrospective setting and no analysis of morbidity and quality of life. However, the intent of this study is to clarify the role of non-curative, resectional surgery in the incurable, asymptomatic AGC, so the quality of life and symptom palliation aren’t the important aspects. What’s more, no vital complications were observed post-operatively.

## Conclusion

There is a role for non-curative surgery plus first-line chemotherapy for incurable asymptomatic AGC in terms of survival and stage, patient age, metastasis type, surgery type should not be limiting factors. The ongoing GYMSSA and REGATTA trials are expected to highlight the value of gastrectomy with metastectomy and systemic therapy versus systemic therapy alone in metastatic gastric cancer. Randomized controlled trials are warranted to fill a gap in knowledge about patient selection strategies.

## Supporting Information

Figure S1 **Kaplan-Meier curves of non-curative surgery group and first-line chemotherapy only group, by surgery types.**
(TIF)

Figure S2 **Kaplan-Meier curves of non-curative surgery group and first-line chemotherapy only group, by patient age.**
(TIF)

Figure S3 **Distribution of the propensity score according to treatment group in entire population.**
(TIF)

Figure S4 **Distribution of the propensity score according to treatment group in stage 4 subpopulation.**
(TIF)

Figure S5 **Kaplan-Meier curves of non-curative surgery group and first-line chemotherapy only group, by baseline serum CEA.** CEA, carcinoembryonic antigen.
(TIF)

Figure S6 **Kaplan-Meier curves of non-curative surgery group and first-line chemotherapy only group, by baseline ascites.**
(TIF)

Table S1 **Univariate analysis of overall survival in patients with advanced gastric cancer.**
(DOC)

Table S2 **Multivariate analysis of overall survival in patients with advanced gastric cancer.**
(DOC)

Table S3 **Multivariate analysis of overall survival in patients with stage 4 gastric cancer.**
(DOC)
