# Supplementary figures and images for: The Role of Non-Curative Surgery in Incurable, Asymptomatic Advanced Gastric Cancer
Source: PLoS One. 2013 Dec 16;8(12):e83921. doi: 10.1371/journal.pone.0083921 (PMC3865283; doi:10.1371/journal.pone.0083921)

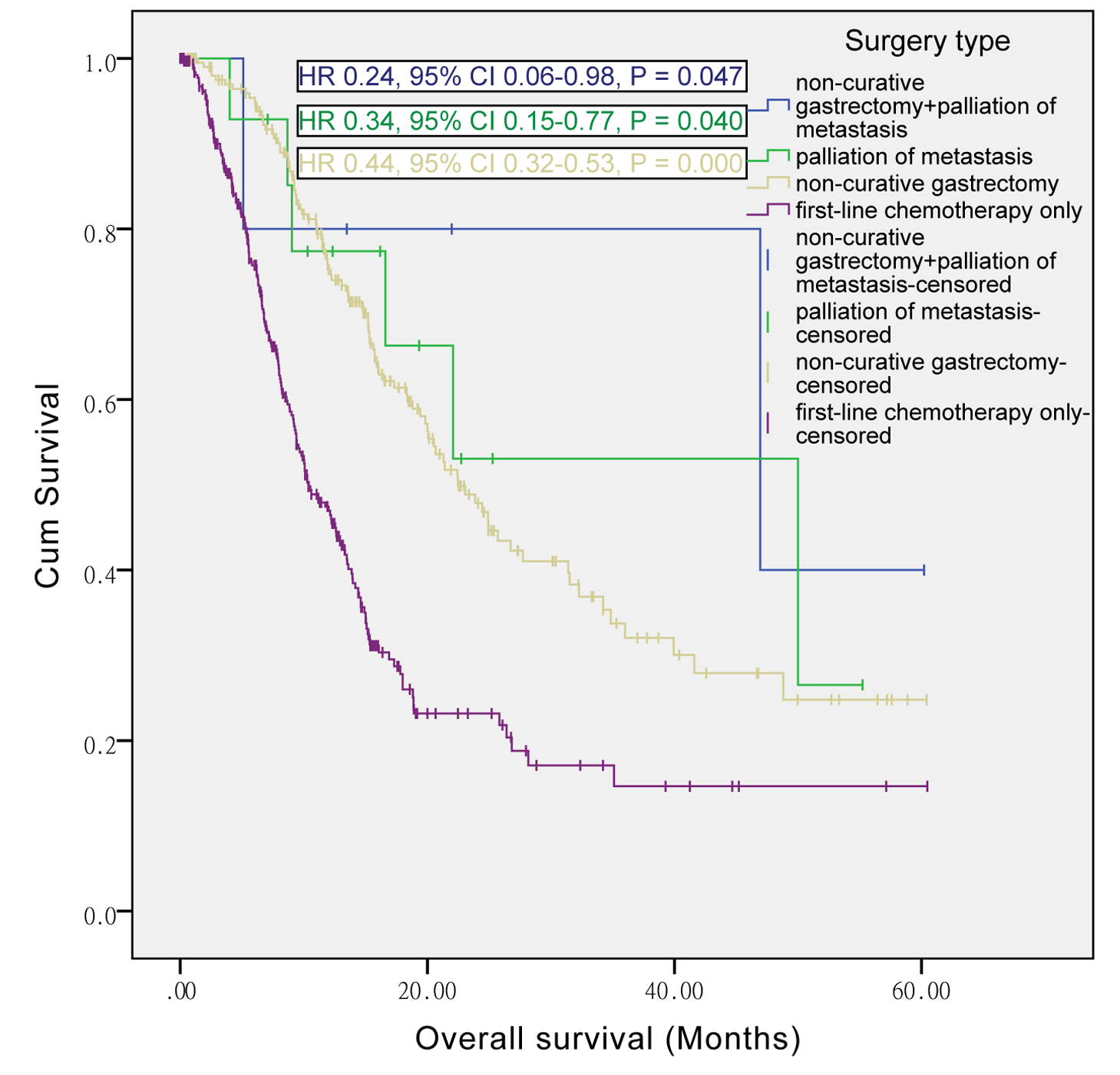

Supplement: Figure S1 — Kaplan-Meier curves of non-curative surgery group and first-line chemotherapy only group, by surgery types. (TIF) [file pone.0083921.s001.tif]

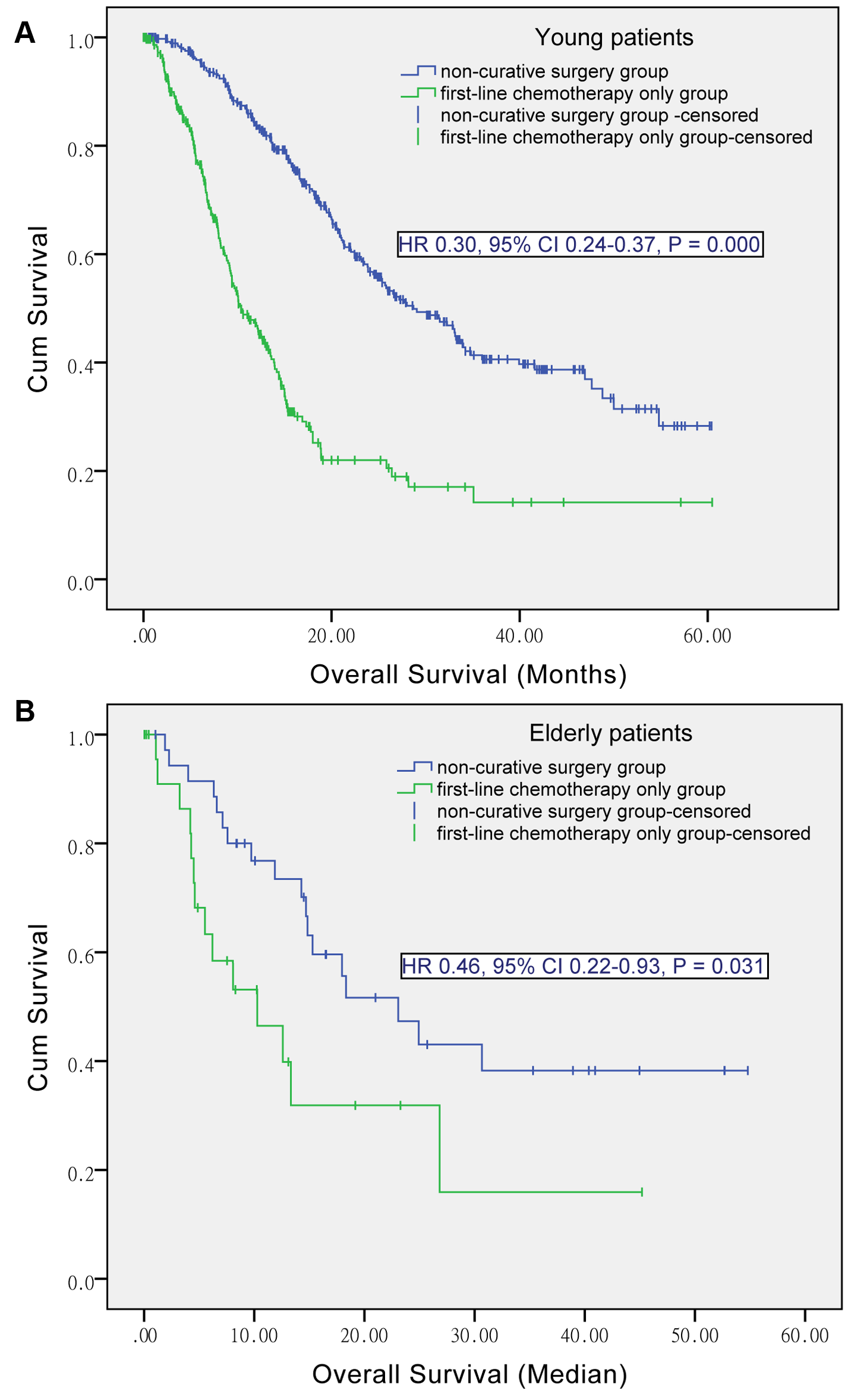

Supplement: Figure S2 — Kaplan-Meier curves of non-curative surgery group and first-line chemotherapy only group, by patient age. (TIF) [file pone.0083921.s002.tif]

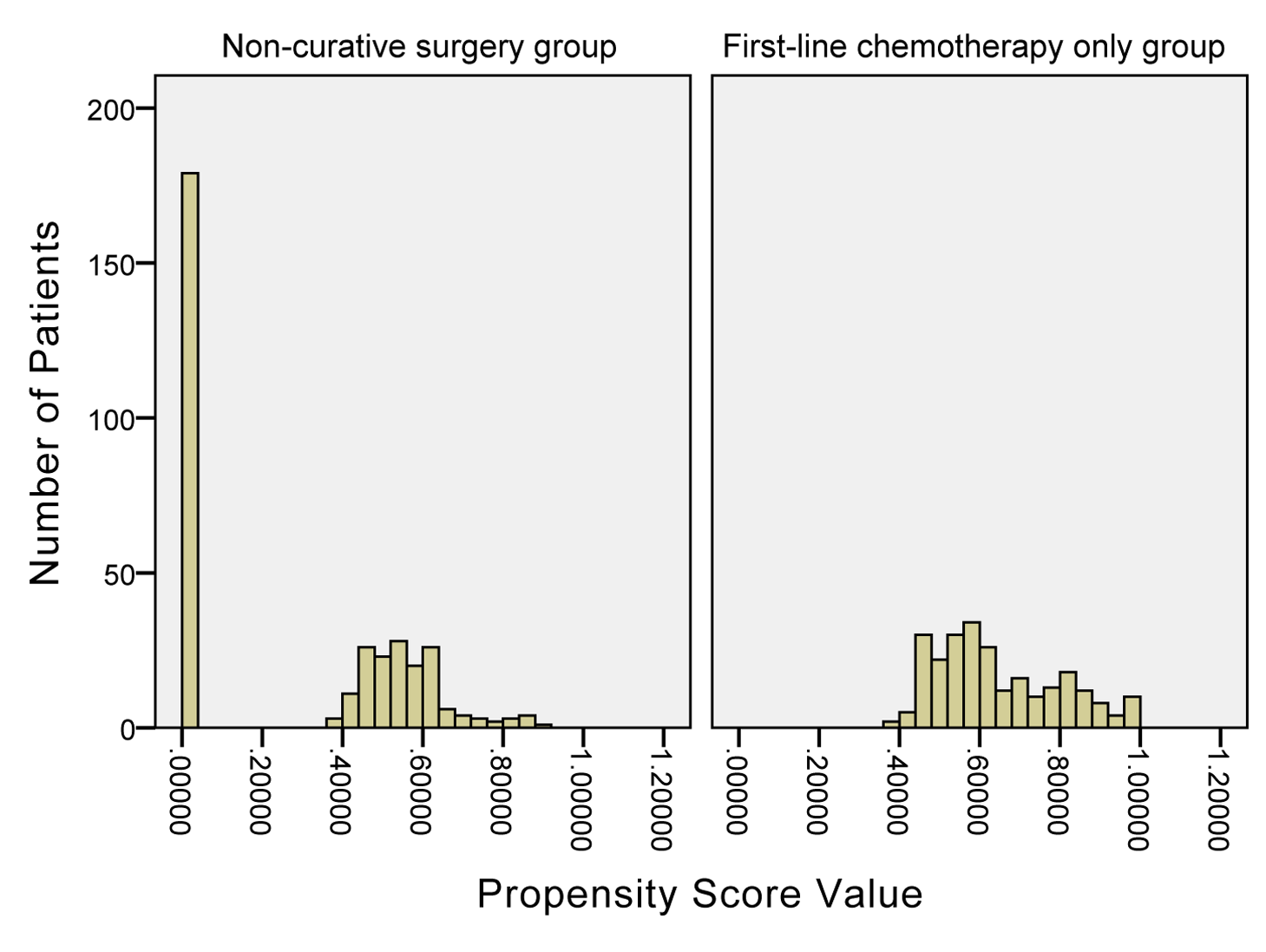

Supplement: Figure S3 — Distribution of the propensity score according to treatment group in entire population. (TIF) [file pone.0083921.s003.tif]

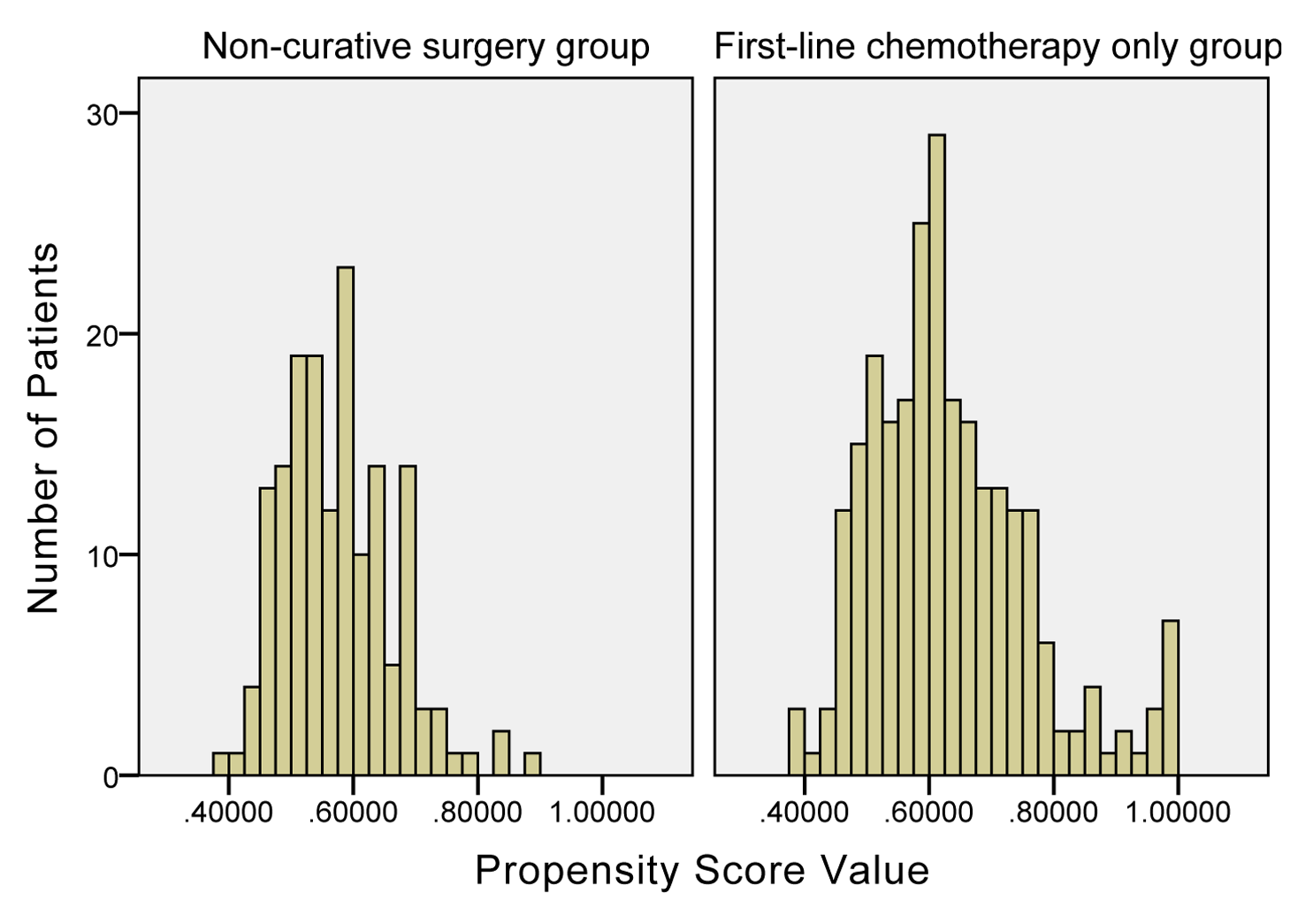

Supplement: Figure S4 — Distribution of the propensity score according to treatment group in stage 4 subpopulation. (TIF) [file pone.0083921.s004.tif]

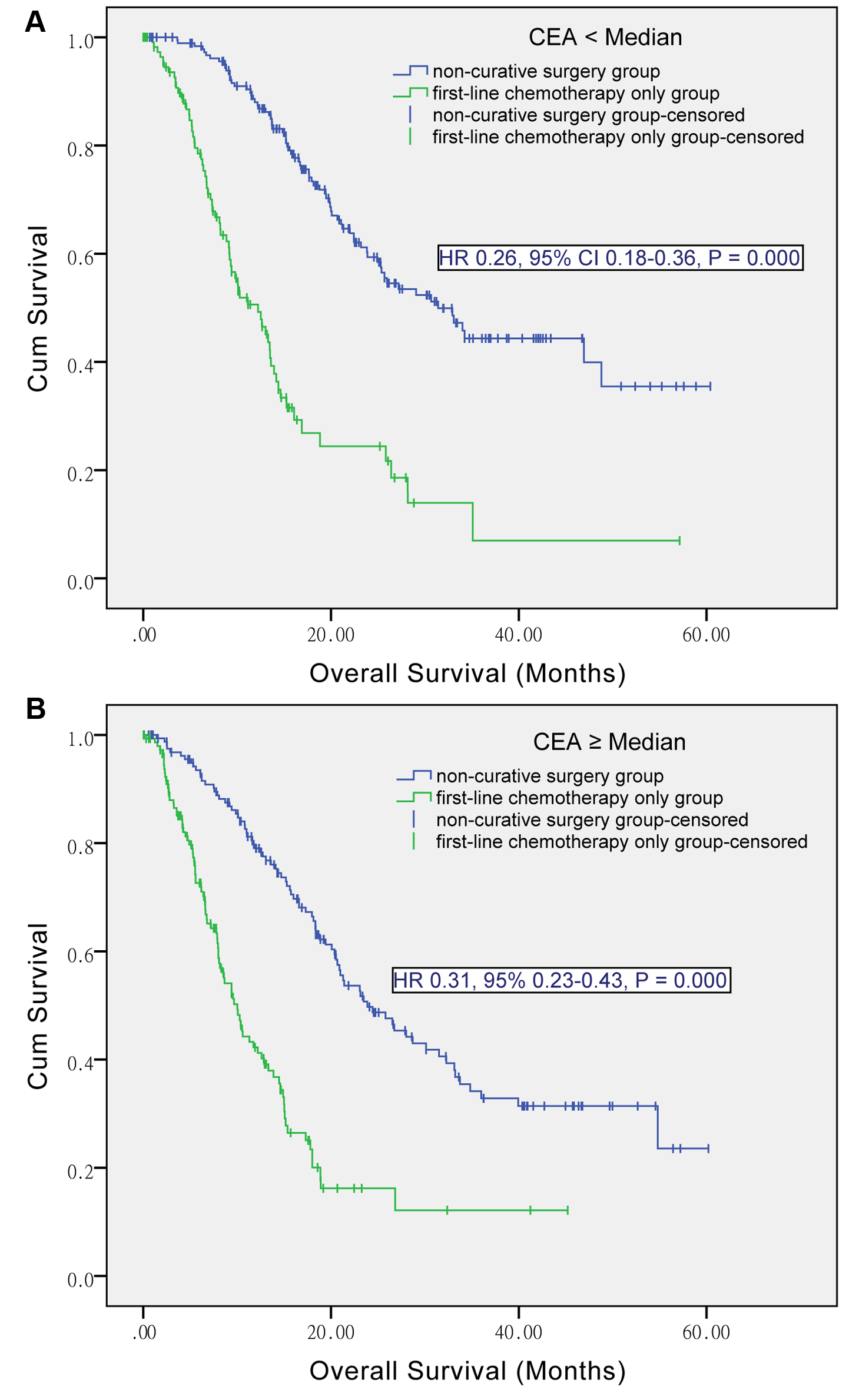

Supplement: Figure S5 — Kaplan-Meier curves of non-curative surgery group and first-line chemotherapy only group, by baseline serum CEA. CEA, carcinoembryonic antigen. (TIF) [file pone.0083921.s005.tif]

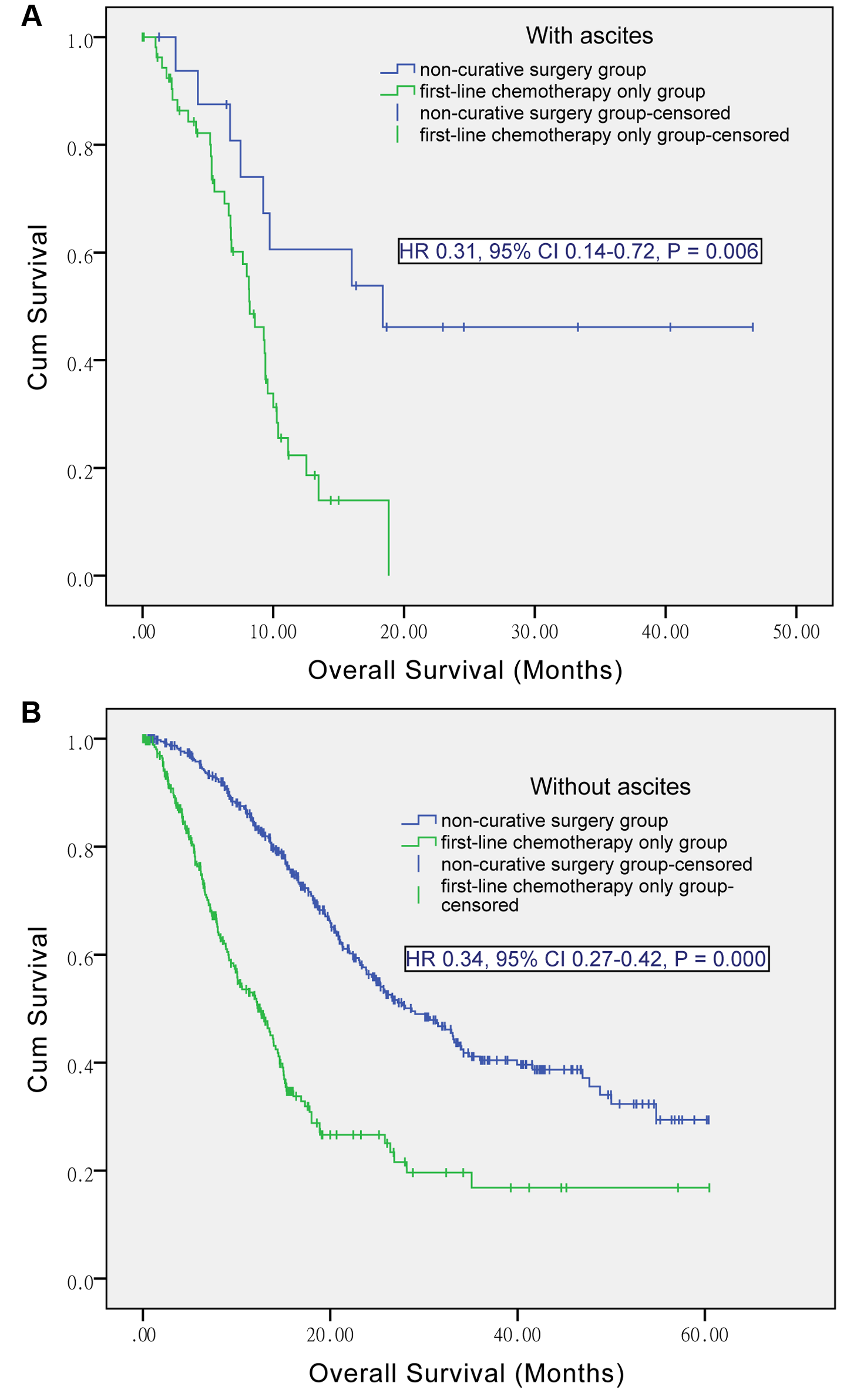

Supplement: Figure S6 — Kaplan-Meier curves of non-curative surgery group and first-line chemotherapy only group, by baseline ascites. (TIF) [file pone.0083921.s006.tif]
