# Supplementary material for: The Role of Non-Curative Surgery in Incurable, Asymptomatic Advanced Gastric Cancer
Source: PLoS One. 2013 Dec 16;8(12):e83921. doi: 10.1371/journal.pone.0083921 (PMC3865283; doi:10.1371/journal.pone.0083921)
Supplement: Table S1 — Univariate analysis of overall survival in patients with advanced gastric cancer. (DOC) [file pone.0083921.s007.doc]

| Variate | n | Median survival (months) | HR | 95% Cl | P-value |
| --- | --- | --- | --- | --- | --- |
| Treatment |  |  |  |  | 0.000 |
| Non-curative surgery+chemotherapy | 414 | 28.00 | 0.31 | 0.25-0.38 |  |
| Chemotherapy only | 323 | 10.37 | 1 | reference |  |
| AJCC stage |  |  |  |  | 0.000 |
| Stage 3 | 191 | 33.13 | 0.40 | 0.31-0.52 |  |
| Stage 4 | 546 | 15.07 | 1 | reference |  |
| Tumor location |  |  |  |  | 0.036 |
| Proximal | 502 | 18.33 | 1.28 | 1.02-1.61 |  |
| Distal | 235 | 25.80 | 1 | reference |  |
| Ascites |  |  |  |  | 0.000 |
| No | 665 | 21.07 | 0.40 | 0.29-0.55 |  |
| Yes | 72 | 9.27 | 1 | reference |  |
| Serum CEA |  |  |  |  | 0.003 |
| < the median | 309 | 22.47 | 0.71 | 0.57-0.89 |  |
| ≥ the median | 311 | 17.30 | 1 | reference |  |
| Serum 199 |  |  |  |  | 0.000 |
| < the median | 296 | 25.80 | 0.59 | 0.47-0.74 |  |
| ≥ the median | 299 | 15.33 | 1 | reference |  |
| Serum 724 |  |  |  |  | 0.000 |
| < the median | 209 | 29.07 | 0.53 | 0.40-0.71 |  |
| ≥ the median | 212 | 17.93 | 1 | reference |  |

**Abbreviations:** HR, hazard ratio; CI, confidence interval; Stage 4, including metastatic and recurrent gastric cancer; CEA, baseline carcinoembryonic antigen; CA19-9, baseline carbohydrate antigen 19-9; CA72-4, baseline carbohydrate antigen 72-4.
