# Supplementary material for: The Role of Non-Curative Surgery in Incurable, Asymptomatic Advanced Gastric Cancer
Source: PLoS One. 2013 Dec 16;8(12):e83921. doi: 10.1371/journal.pone.0083921 (PMC3865283; doi:10.1371/journal.pone.0083921)
Supplement: Table S2 — Multivariate analysis of overall survival in patients with advanced gastric cancer. (DOC) [file pone.0083921.s008.doc]

**Table** **S2.** Multivariate analysis of overall survival in patients with advanced gastric cancer.

| Variate | HR | 95% Cl | P |
| --- | --- | --- | --- |
| Treatment |  |  | 0.000 |
| Non-curative surgery+chemotherapy | 0.36 | 0.26-0.52 |  |
| Chemotherapy only | 1 | reference |  |
| AJCC stage |  |  | 0.006 |
| Stage 3 | 0.60 | 0.42-0.86 |  |
| Stage 4 | 1 | reference |  |
| Tumor location |  |  | 0.026 |
| Proximal | 1.44 | 1.05-1.98 |  |
| Distal | 1 | reference |  |
| Ascites |  |  | 0.020 |
| No | 0.58 | 0.37-0.92 |  |
| Yes | 1 | reference |  |
| Serum CEA |  |  | 0.043 |
| < the median | 0.74 | 0.56-0.99 |  |
| ≥ the median | 1 | reference |  |

**Abbreviations:** HR, hazard ratio; CI, confidence interval; AJCC, American Joint Committee on Cancer; Stage 4, including metastatic and recurrent gastric cancer; CEA, baseline carcinoembryonic antigen.
