# Supplementary material for: The Role of Non-Curative Surgery in Incurable, Asymptomatic Advanced Gastric Cancer
Source: PLoS One. 2013 Dec 16;8(12):e83921. doi: 10.1371/journal.pone.0083921 (PMC3865283; doi:10.1371/journal.pone.0083921)
Supplement: Table S3 — Multivariate analysis of overall survival in patients with stage 4 gastric cancer. (DOC) [file pone.0083921.s009.doc]

**Table S3.** Multivariate analysis of overall survival in patients with stage 4 gastric cancer.

| Variate | HR | 95% Cl | P |
| --- | --- | --- | --- |
| Treatment |  |  | 0.000 |
| Non-curative surgery+chemotherapy | 0.35 | 0.24-0.50 |  |
| Chemotherapy only | 1 | reference |  |
| Ascites |  |  | 0.017 |
| No | 0.56 | 0.35-0.90 |  |
| Yes | 1 | reference |  |
| Serum CEA |  |  | 0.027 |
| < the median | 0.67 | 0.47-0.96 |  |
| ≥ the median | 1 | reference |  |
| Serum CA19-9 |  |  | 0.028 |
| < the median | 0.68 | 0.48-0.96 |  |
| ≥ the median | 1 | reference |  |

**Abbreviations:** Stage 4, including metastatic and recurrent gastric cancer; HR, hazard ratio; CI, confidence interval; CEA, baseline carcinoembryonic antigen; CA19-9, baseline carbohydrate antigen 19-9.
